# Oral and Topical *Centella asiatica* in Type 2 Diabetes Mellitus Patients with Dry Skin: A Three-Arm Prospective Randomized Double-Blind Controlled Trial

**DOI:** 10.1155/2020/7253560

**Published:** 2020-08-26

**Authors:** Lili Legiawati, Kusmarinah Bramono, Wresti Indriatmi, Em Yunir, Siti Setiati, Sri Widia A. Jusman, Erni H. Purwaningsih, Heri Wibowo, Retno Danarti

**Affiliations:** ^1^Department of Dermatology and Venereology, Faculty of Medicine, Universitas Indonesia, Jakarta 10430, Indonesia; ^2^Department of Internal Medicine, Faculty of Medicine, Universitas Indonesia, Jakarta 10430, Indonesia; ^3^Department of Biochemistry and Molecular Biology, Faculty of Medicine, Universitas Indonesia, Jakarta 10430, Indonesia; ^4^Department of Medical Pharmacy, Faculty of Medicine, Universitas Indonesia, Jakarta 10430, Indonesia; ^5^Integrated Laboratorium, Faculty of Medicine, Universitas Indonesia, Jakarta 10430, Indonesia; ^6^Department of Dermatology and Venereology, Faculty of Medicine, Public Health, and Nursing, Universitas Gadjah Mada, Yogyakarta 55281, Indonesia

## Abstract

**Introduction:**

Uncontrolled diabetes mellitus (DM) is related to skin disorders, particularly dry skin. Pathogenesis of dry skin in type 2 diabetes mellitus (T2DM) rises from the chronic hyperglycemia causing an increase in *advanced glycation end-products* (AGEs), proinflammatory cytokines, and oxidative stress. Combination of oral and topical *Centella asiatica* (CA) is expected to treat dry skin in T2DM patients more effectively through decreasing N(6)-carboxymethyl-lysine (CML) and interleukin-1*α* (IL-1*α*) and increasing superoxide dismutase (SOD) activity.

**Methods:**

A three-arm prospective, double-blind, randomized, controlled study was performed to evaluate the efficacy of the oral and topical CA extract in 159 T2DM patients with dry skin. The subjects were divided into the CA oral (CAo) 2 × 1.100 mg + CA topical (CAt) 1% ointment group, oral placebo (Plo) + CAt group, and Plo and topical placebo (Plt) group. Dry skin assessment was performed on day 1, 15, and 29, while evaluation of CML, IL-1*α*, and SOD activity was on day 1 and 29.

**Result:**

Effectivity of CAo + CAt combination was assessed based on HbA1c and random blood glucose (RBG). In well-controlled blood glucose, on day 29, the percentage of SRRC decrement was greater in the CAo + CAt group compared to the control group (*p* = 0.04). SCap value in the CAo + CAt group was greater than that in the control group (*p* = 0.01). In the partially controlled blood glucose, increment of SOD activity in the CAo + CAt group was greater than that in the control group (*p* = 0.01). There were medium-to-strong correlation between CML with SOD (*r* = 0.58, *p* < 0.05) and IL-1*α* with SOD (*r* = 0.70, *p* < 0.05) in well-controlled blood glucose. Systemic and topical adverse events were not significantly different between groups.

**Conclusion:**

CAo and CAt combination can be used to significantly improve dry skin condition through increasing SOD activity in T2DM patients with controlled blood glucose.

## 1. Introduction

Diabetes mellitus (DM) is a metabolic disease caused by insulin resistance or defect of insulin secretion resulting in hyperglycemia. There are several types of diabetes, with 90% of the cases being T2DM. In T2DM, peripheral tissue insulin sensitivity and *β*-cell pancreas insulin secretion are disrupted. Increasing incidence of diabetes creates a growing healthcare financial burden in Indonesia as it is expected to cost 1.2 million US dollar by 2020 [1, 2].

Uncontrolled DM is highly correlated with various chronic complications, and amongst them is skin complication. Chatterjee et al. [3] found that 74% of T2DM patients had one or more skin problems, with the highest amount being dry skin (47%), followed by infection (10%), diabetic foot (5%), hair loss, and diabetic dermopathy (4%), respectively. Although skin problems are often found in T2DM patients, the awareness and choice of treatment are commonly lacking. Dry skin treated properly in early stages can lower diabetes patients' morbidities and prevent complications, which includes infection, ulcer, and gangrene, which could result in amputation [4].

Diabetes mellitus affects the skin with several mechanisms where hyperglycemia and advanced glycation end-products (AGEs) are the two main factors. The pathologic effect of AGEs is caused by its ability in inducing oxidative stress and inflammation through binding with the receptor on the cell surface and body protein cross-linking, thus altering the cell structure and function. Concentration of AGEs in diabetes patients is significantly higher due to the hyperglycemic state and oxidative stress. One commonly known AGEs used as a parameter, N(6)-carboxymethyl-lysine (CML), was increased in DM patients. The study showed that CML was detected in the skin epidermal layer [5]. Aside from AGEs and oxidative stress, DM is correlated with the disruption of anti-inflammatory response in which cytokine plays a key role [6].

*Centella asiatica* (CA) or commonly known as *pegagan* in Indonesia is widely used as a traditional remedy, and its benefits had been scientifically proven [7]. There are several studies concerning the benefits of CA in topical and oral regiment. Topical CA has low bioavailability, but research has found that topical CA can increase skin hydration by expressing aquaporin-3 (AQP-3), humectant, anti-inflammation, antiglycation, and antioxidant [8]. However, for oral CA, Yuan et al. stated that CA consumption in rats showed a systemic bioavailability of 16.25%. Even though the oral CA bioavailability is low, other research studies have proven that oral CA has antidiabetic, antidepressant, antibacterial, wound healing, and neuroprotective effects [7]. Jenwitheesuk et al. [9] studied that 7% of *Centella asiatica* cream application in split-thickness skin graft (STSG) patients showed better scar development. Combination of CA and *P. amboinicus* cream for 14 days improved diabetic foot ulcer, which was comparable to the hydrocolloid fiber dressing without significant difference in effectiveness [10].

From the authors' perspective, the addition of CAo into CAt is expected to be able to resolve the systemic factor related to the pathogenesis of dry skin in DM, thus increasing its effectiveness in treating dry skin. *Centella asiatica* also has its own potential for treating dry skin in DM with regard to the multiple attributes of CA, including anti-inflammation, antioxidant, and other effects. Based on the literature search from PubMed, Cochrane, and Google Scholar, a similar research has not been conducted yet. Compared to other studies about CA, this study evaluated the combination of oral and topical CA compared to topical CA only and the control without CA regiment for treating dry skin in T2DM patients. This study also aimed to investigate the CA's mechanism of action in repairing dry skin in T2DM patients through AGEs CML, interleukin-1*α*, and SOD.

## 2. Materials and Methods

### 2.1. Design Overview

This research was a double-blind randomized clinical trial in T2DM patients with dry skin intended to assess CA's effect in improving dry skin. Improvement was clinically measured by specific symptom sum score (SRRC) and skin capacitance (SCap), and then the cellular pathway was investigated by measuring the concentration of AGEs CML, IL-1*α*, and SOD in the stratum corneum.

### 2.2. Setting and Participants

The subjects were patients with T2DM enrolled from the metabolic endocrine outpatient clinic—Dr. Cipto Mangunkusumo National Central General Hospital—and 5 primary healthcare facilities in Jakarta. Research was conducted in the dermatovenereology outpatient clinic in the same hospital with the duration of 8 months from July 2018 to March 2019. The study was approved by the Health Research Ethics Committee, Faculty of Medicine, Universitas Indonesia, and Dr. Cipto Mangunkusumo National Central General Hospital. This study was also registered in clinicaltrials.gov.

Inclusion criteria were T2DM patients with dry skin on low extremities evaluated by SRRC with a score above 3, were under the age of 60 years, were willing to not use any skin care products, both oral and/or topical, on low extremities or other medications beside DM treatment, had a normal ankle brachial index (ABI) score (ranging from 0.9 to 1.3), and indicated that they were willing to follow the research instruction by signing the informed consent form. Exclusion criteria were patients with diabetic ulcer, infection, and/or dermatitis on one of the lower extremities, with severe inflammation with an erythema score above 3 and/or a fissure score above 3 on the SRRC and the SRRC score is between 12 and 16, glomerular filtration rate (GFR) < 45, and liver function abnormalities. Patients were dropped out from the study if during the study, the patients experienced severe side effect, digestive and liver function abnormality, and severe hypoglycemia symptoms, patients resigned from the study, and patients did not follow the study appointed protocol.

### 2.3. Subjects

Research subjects consisted of 53 subjects in each treatment group, a total of 159 subjects. The sample size was calculated using the formula for proportion and correlation study.

### 2.4. Randomization and Interventions

**Figure 1:**
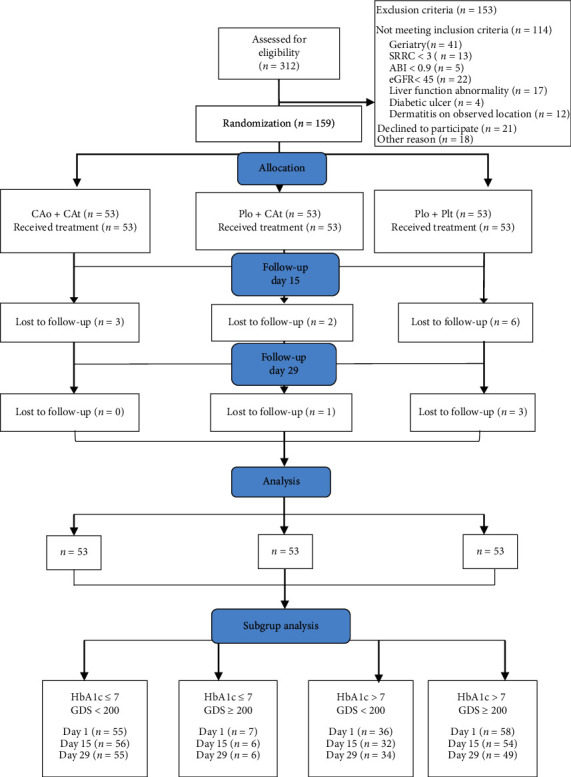
CONSORT flow diagram.

Randomization was conducted by research supervisors using a code or a password for each subject with a computer program (Randlist®). After doing the preliminary examination, the subjects were divided into three groups: the first group was treated with CAo with the dose of 2 × 1100 mg and CAt 1% ointment, the second group treated with oral placebo (Plo) and CAt 1% ointment, and the third group treated with Plo and topical placebo (Plt), vaseline album. Oral therapies were consumed by the participant twice daily, and topical therapies were also applied twice a day. Subjects were instructed to apply 0.25 g topical ointment on the right lower extremities using a transparent imprint size 7 × 20 cm. All subjects still received routine DM medications. All three groups underwent interventions for 28 days. Each patient was scheduled for follow-up at day 15 and day 29 (Figure 1).

### 2.5. Formulation of Investigational Product

*Centella asiatica* ingredient used in this research was from Tawangmangu, Central Java, Indonesia. The oral CA and placebo were produced by an international standardized pharmaceutical company, while CA ointment and placebo ointment were produced by the Pharmacy Department, Faculty of Medicine, Universitas Indonesia. Both oral CA and oral placebo were produced in a similar capsule and unscented, while both ointment, the active and the placebo, were packaged with the same tube packagings.

### 2.6. Skin Stripping

The technique for stratum corneum extraction was done by the skin-stripping technique modified from the study by Perkins et al. [11]. This study used cyanoacrylate skin surface stripping (CSSS) from 3S-Biokit®. We altered the procedure by using a transparent foil of 3.75 cm × 2.5 cm with 20 *μ*L cyanoacrylate adhesive. The adhesive was applied for 5 minutes before harvested. In the laboratory, the samples were later processed into the homogenate form in less than 2 hours. Evaluation for CML was done using the ELISA kit from MyBioSource®, IL-1*α* was done using the ELISA kit from *Cloud-Clone Corp*®, and SOD was done using the RANSOD® kit produced by RANDOX Laboratories®.

### 2.7. Outcomes and Measurements

Patient demographic data were recorded. Pruritus and pain were measured by using the visual analog scale (VAS), and the score was classified to no pruritus/pain (0), mild (1–3), moderate (4–6), and severe (7–10). Neuropathy was assessed by using the survey of autonomic symptoms (SAS). Laboratory examination was performed for the HbA1c and triglyceride on day 1, the liver function measuring both SGOT (serum glutamic oxaloacetic transaminase) and SGPT (serum glutamic pyruvic transaminase) on days 1 and 29, and the random blood glucose (RBG) on days 1, 15, and 29. Blood sampling for RBG, SGOT, SGPT, and triglyceride was done using the 3 ml vein blood sample collected in a nonanticoagulated tube; meanwhile, for HbA1c, it was done using the 3 ml vein blood sample collected in an ethylenediaminetetraacetic acid (EDTA) tube. Clinical examination of the dry skin was conducted by using the SRRC that measures the scaling, roughness, redness, and crack fissure. The SCap was measured by using the corneometer in arbitrary unit (AU). The corneometer used was corneometer CM825®, which was manufactured by Courage-Khazaka, Germany. Skin samples taken from the skin-stripping technique were processed into a homogenate. The homogenate was then tested to measure the CML, IL-1*α*, and SOD concentration.

### 2.8. Statistical Analysis

The data were saved in a database and then processed with SPSS v.24 and Stata v.14 (StataCorp). The analysis conducted was *intention to treat* and per protocol. The chi-square test was applied to population data with nominal data. The continuous data were analyzed with the Kruskal–Wallis test amongst the three groups, followed by the Mann–Whitney test between each group. A correlation study was conducted with the Spearman test. If the *p* value was <0.05, the result was considered to be statistically significant.

## 3. Results

### 3.1. Sample Characteristics

In total, 312 patients were screened, in which 159 met the inclusion and exclusion criteria and then were randomized and treated. Only 144 of the 159 patients completed the study protocol. There were six patients lost to follow-up, four patients retreated due to occupation related matter, and five patients dropped out due to adverse event.

All the treatment groups had comparable baseline characteristics. There was no significant statistical difference (Table 1), except for the subjects' age. The median subject age was however ranging narrow between 52 and 54 years. Most of the subjects were females (75.4%) who were mainly housewives (59.7%). Subjects' education level was majority intermediate level, graduated from middle or high school (64.1%).

In this study, the majority of subjects had no prior dry skin treatment (71.6%). As shown in Table 1, we found the highest random blood glucose (RBG) and HbA1c on day 1 in Plo + CAt. This group also presented with the highest triglyceride level. The difference was statistically insignificant amongst the three study groups. Nonetheless, the median HbA1c level for the three groups was above 7%, indicating poor blood glucose control across all groups. Specifically, in the CAo treatment group, we observed a declining trend of RBG from day 1, day 15, to day 29, albeit not statistically significant. In the CAo + CAt group, both SGOT and SGPT were not increased from days 1 to 29. The median SGOT values were 18 (range: 9–48) U/L on day 1 and 16 (range: 10–41) U/L on day 29. Meanwhile, the median SGPT values were 21 (range: 7–70) U/L on day 1 and 21 (10–53) U/L on day 29. There were also an insignificant difference of SGOT (*p*=0.73) and SGPT (*p*=0.64) between the CAo + CAt group and the other 2 groups on day 29.

### 3.2. SRRC and SCap

We observed that SRRC reduction (Table 2) in CAo + CAt was higher only when compared to the Plo + CAt group, and the difference was statistically insignificant on day 15 (*p*=0.48) in the three groups. On day 29, the CAo + CAt group did not attain better SRRC reduction compared to other groups, and no significant difference was found amongst them (*p*=0.71). Meanwhile, SCap improvement was found to be the highest in CAo + CAt on day 15 (*p*=0.38). SCap of CAo + CAt on day 29 was higher compared to Plo + Plt but lower than Plo + CAt (*p*=0.39).

### 3.3. CML, IL-1*α*, and SOD Activity

In this study, the value of CML, IL-1*α*, and SOD activity was measured on day 1 and day 29. From the measurement on day 1, CML and IL-1*α* levels were the highest in the Plo + Plt group and SOD activity measured was the highest in the CAo + CAt group. However, the difference in baseline was statistically insignificant between the three groups. On day 29, we found that CML and IL-1*α* levels in the CAo + CAt group were not lower than that in the other groups. The reduction of CML and IL-1*α* levels in the CAo + CAt group was also not superior to the other groups. The SOD activity was found to be higher in the CAo + CAt group (median 5.9 U/mg protein, range 1–59.4 U/mg protein) with the highest value of SOD activity improvement (median 0.9 U/mg protein, range −39.2–20.1 U/mg protein). However, the differences between each group were not statistically significant.

### 3.4. Subgroup Analysis

From our first analysis, the clinical improvement of dry skin was found to be insignificant in the CAo + CAt group. We considered several factors that might affect our study results, which were (1) the medians of HbA1c levels in all three groups were in an uncontrolled level (higher than 7, range 7.4–8); (2) SRRC could not accurately reflect the clinical improvement because when SRRC was scored 0 on day 15, the clinical improvement which happened between day 15 and day 29 would still be scored as 0 on day 29; (3) RBG influence on the body metabolism. Therefore, we performed subgroup analysis based on the blood glucose control, HbA1c, and RBG.

Subgroup analysis was applied to this study due to a wide range of HbA1c of the subjects and a median of HbA1c value >7 although the limit from the Indonesia Clinical Practical Guideline for controlled diabetes was HbA1c ≤ 7% and RBG < 200 mg/dL [12]. Subgroup analysis was conducted to analyse blood glucose effects (HbA1c and RBG) on the parameters: SRRC, SCap, CML, IL-1*α*, and SOD. We analyzed the combination criteria of HbA1c and RBG into HbA1c ≤ 7% and RBG <200 mg/dL (well-controlled blood glucose), HbA1c ≤ 7% and RBG ≥ 200 mg/dL (fairly controlled blood glucose), HbA1c > 7% and RBG < 200 mg/dL (partially controlled blood glucose), and HbA1c > 7% and RBG ≥ 200 mg/dL (poorly controlled blood glucose). However, the fairly controlled subgroup had fewer samples compared to other variants, and even on day 1, there were no Plo + Plt samples; thus, this criterion was not analyzed further in this study.

In SRRC and SCap analysis, we found that in well-controlled blood glucose subgroup (Table 3), both SRRC and SCap of the CAo + CAt group were better among the intervention groups. The SRRC median of the CAo + CAt group was the lowest yet statistically insignificantly differed compared to that of the Plo + Plt group (*p*=0.06). However, the SCap median of the CAo + CAt group was the highest among all the three groups (*p*=0.03), and it was statistically significantly different compared to that of the Plo + Plt group (*p*=0.01). The significant increment in the SCap median was not in line with the insignificant decrement in SRRC; hence, we analyzed the SRRC based on the percentage of SRRC decrement. The CAo + CAt group showed the highest SRRC decrement (median 75%, range 16.7–100%), and it was statistically significant (*p*=0.04) compared to that of the Plo + Plt group. In partially and poorly controlled blood glucose subgroups, we did not find a significant difference of SRRC and SCap between the CAo + CAt group and other intervention groups on day 29.

**Figure 2:**
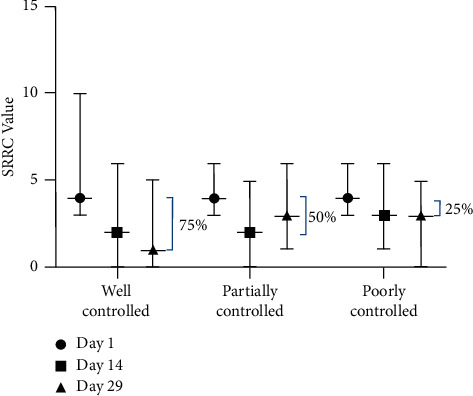
SRRC value of the CAo + CAt treatment group in various blood glucose control subgroups.

From the beginning of the treatment, the median of SRRC of each blood glucose control (Figure 2) was the same. However, the well-controlled blood glucose subgroup had a wider range (median 4, range 3–10). When we compared SRRC median reduction in various HbA1c and RBG levels, SRRC for CAo + CAt in the well-controlled blood glucose subgroup decreased by 75% compared to that of the partially controlled blood glucose subgroup (50%) and the poorly controlled blood glucose subgroup (25%). The median reduction of the well-controlled blood glucose subgroup continued to decrease until day 29, while it remained the same for poorly controlled blood glucose from day 15 to day 29, and for the partially controlled blood glucose subgroup, it slightly increased on day 29.

**Figure 3:**
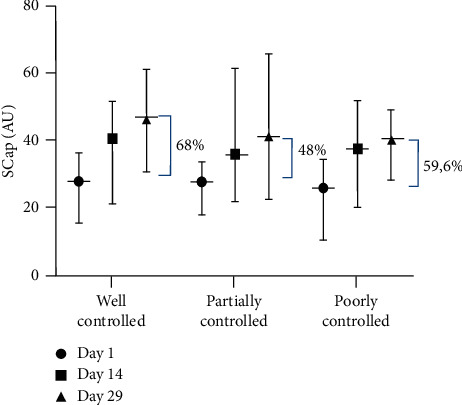
SCap improvement of the CAo + CAt treatment group in various blood glucose control subgroups.

The baseline value of SCap was found to be almost similar in the CAo + CAt group amongst various blood glucose controls (Figure 3). SCap improvement in the well-controlled blood glucose subgroup was measured to be about 68% compared to that of the partially controlled blood glucose subgroup (48%) and the poorly controlled blood glucose subgroup (59.6%). The alignment of SCap improvement in the well-controlled blood glucose subgroup seemed more inclined compared to the other two groups.

In the analysis of the well-controlled blood glucose subgroup, we found that CML and IL-1*α* levels in the CAo + CAt group were not lower compared to that of other groups on day 29. SOD activity was found to improve upmost in the CAo + CAt group. Regardless, the CML, IL-1*α*, and SOD activity were not statistically significant in the well-controlled blood glucose subgroup.

Although the partially controlled blood glucose subgroup showed an almost similar result (Table 4) where there was no reduction in CML and IL-1*α* activity in the CAo + CAt group on day 29, the SOD activity increased significantly higher than that of the other groups (*p*=0.03). We performed the Mann–Whitney test to compare each study group. SOD activity of the CAo + CAt group was significantly higher compared to that of the Plo + Plt group (*p*=0.01).

**Figure 4:**
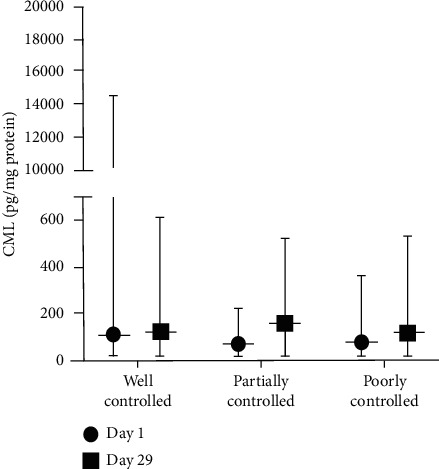
CML level of the CAo + CAt treatment group in various blood glucose control subgroups.

In Figure 4, all CML levels in the CAo + CAt group amongst various blood glucose controls increased on day 29 compared to day 1. The level of increment in the well-controlled blood glucose subgroup was lower compared to that in the other two groups.

**Figure 5:**
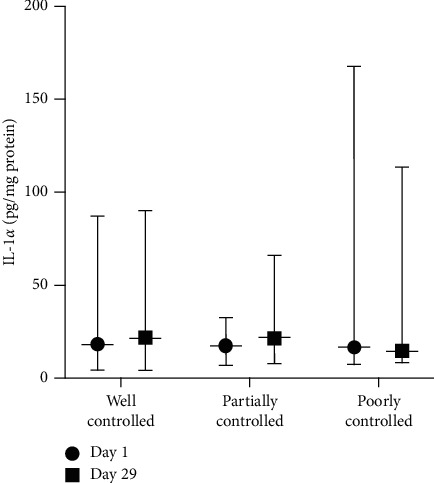
IL-1*α* level of the CAo + CAt treatment group in various blood glucose control subgroups.

Figure 5 shows the IL-1*α* level of the CAo + CAt group in various blood glucose controls. On day 29, there was a reduction in the IL-1*α* level only in the poorly controlled blood glucose subgroup. Meanwhile, in the well-controlled blood glucose subgroup and the partially controlled blood glucose subgroup, the IL-1*α* level was slightly increased.

**Figure 6:**
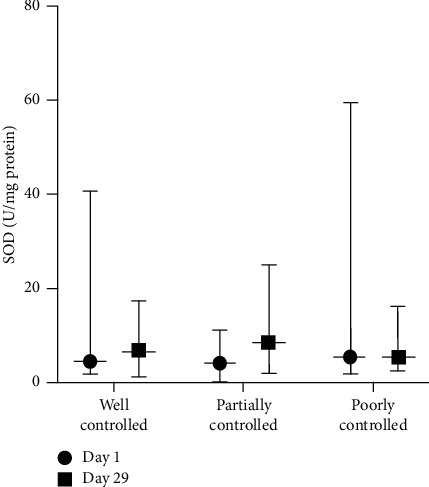
SOD activity of the CAo + CAt treatment group in various blood glucose control subgroups.

The SOD activity median value of the CAo + CAt group in various blood glucose control (Figure 6) baseline was comparable. SOD activity elevation on day 29 was found in the well-controlled blood glucose subgroup and the partially controlled blood glucose subgroup. The elevation in the partially controlled blood glucose subgroup (115%) was higher compared to that of the well-controlled blood glucose subgroup (55%). In the poorly controlled blood glucose subgroup, there was a 10% reduction in SOD activity.

### 3.5. Correlation Analysis

**Figure 7:**
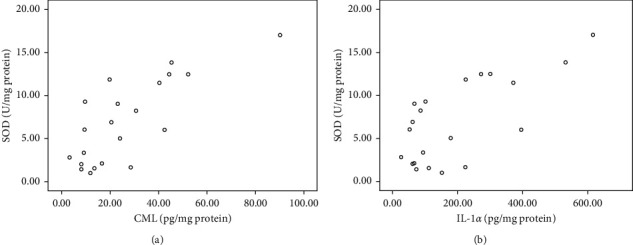
Scatter diagram showing relationships between (a) SOD activity versus CML level and (b) SOD activity versus IL-1*α* level in CAo + CAt with the well-controlled blood glucose group.

At first, we tried to perform correlation analysis between the decreased SRRC and increased SCap with the decreased CML, decreased IL-1*α*, and increased SOD activity in various blood glucose control subgroups. There was no significant correlation in the analysis. Despite no significant correlation between clinical and molecular parameters, we performed correlation analysis between CML and IL-1*α* levels with SOD activity in various blood glucose control subgroups to investigate the relation between molecular parameters. There was no correlation found in partially and poor-controlled blood glucose subgroups, but we found correlation of both CML and IL-1*α* levels with SOD activity in the CAo + CAt group with the well-controlled blood glucose group (Figure 7). Increased CML level was moderately correlated with the increased SOD activity (*r* = 0.58, *p*=0.01). Strong correlation was also found between increased IL-1*α* and increased SOD activity (*r* = 0.70, *p* < 0.001).

### 3.6. Adverse Events

Both oral and topical adverse events were assessed in this study. Each subject could experience more than one adverse event. Oral adverse events, including gastrointestinal symptoms, sedation, increase in urination, abdominal pain, back pain, erectile dysfunction, hoarse voice, vertigo, palpitation, recurring urticaria, herpes zoster, Bell's palsy, and vaginal bleeding, were found to be indifferent between three groups. Gastrointestinal problems were the most common adverse events in all groups with the incidence ranging from 15.8 to 20.1%. Topical adverse events found were burning sensation, tenderness, and mild pruritus; however, there was no significant difference among all treatment groups. We also evaluated SGOT and SGPT on days 1 and 29 which were not increased or differed significantly among the three groups.

## 4. Discussion

The majority of this study's subjects were females with a total of 120 subjects (75.4%). In Indonesia, based on the national data in 2018, DM patients were mainly females (1.8%), compared to males (1.2%) [13]. In this study, females were mainly enrolled because the subjects from 5 primary healthcare facilities were a part of a long-term diabetic surveillance program where most females with a nonstrict daily schedule were willing to visit for follow-up. The median age was 52–54 years with patients aged over 60 years excluded from the study. Therefore, this study result is slightly different from Indonesia's national survey which showed that the most prevalent DM patients are older between 55 and 64 years (6.3%) [14]. The majority of the subjects in this study (73.1%) never had dry skin treatment before with a median duration of dry skin condition of 12 months in each intervention group.

Before performing subgroup analysis, there were no significant improvement of both SRRC and SCap in the CAo + CAt group on day 29. Analysis of CML, IL-1*α*, and SOD showed that the CML and IL-1*α* were not lower in CAo + CAt on day 29 where SOD activity was higher but statistically insignificant on day 29. We presumed a factor of wide range of HbA1c, while the median HbA1c value all of the groups was >7 (controlled diabetic has HbA1c value ≤ 7), affecting the result of this study. As in previous studies, glycemic control strongly influences dry skin where HbA1c strongly correlates with the prevalence of dry skin [15, 16], while RBG influences the body metabolism [17]. Therefore, a subgroup analysis was conducted by distinguishing the glycemic control level of both HbA1C and RBG. Subgroup analysis was performed to observe whether the various HbA1c and RBG levels influenced the result's significance.

**Figure 8:**
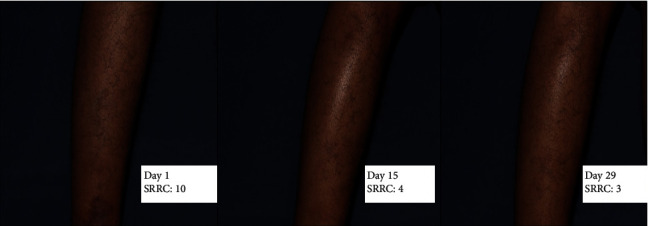
The improvement of the SRRC score in the CAo + CAt intervention group through days 1, 15, and 29.

In the well-controlled blood glucose subgroup, the percentage of SRRC reduction (Figure 8) and SCap improvement was significant in the CAo + CAt group. This difference was not found in the other subgroups, thus indicating the influence of HbA1c and RBG on SRRC and SCap. However, long-term control measured by the HbA1c level had a greater influence compared to the RBG level. Controlled RBG level was too fluctuating and too short to provide adequate time for SRRC reduction and SCap improvement. Moreover, CAo + CAt from two groups with HbA1c > 7 only showed SRRC reduction until day 15, and between day 15 and day 29, the graph showed a stagnant reduction. A previous study also mentioned correlation between HbA1c with SRRC and SCap [18]. There was only limited studies discussing CA therapy in dry skin, and none of currently available studies performed combination of oral and topical CA. In an in vitro study by Wijayadi et al. [8], it was found that CA enhances skin hydration by inducing the expression of aquaporin-3 (AQP-3). One study comparing the CA extract in nanoparticle chitosan (CAEENPK), CA extract in ethanol (CAE), and standard moisturizer in dry skin of elderly showed clinical improvement in the CA-treated group measured by SRRC and SCap [19]. On day 15, SCap of the CAo + CAt group increased higher than that of other groups although it was statistically insignificant where SRRC of the CAo + CAt group did not decrease lower than the other groups. This result showed a pattern where SCap improvement would prevail the SRRC improvement, meaning that dry skin restoration happened after the restoration of the water-binding ability of the stratum corneum. Guzmán-Alonso's study [20] stated that there was a correlation between lack of water-binding ability of the stratum corneum which caused a water barrier disruption with the result of difficulty in reaching dry skin restoration.

When we compared the well-controlled with the partially controlled blood glucose group in CAo + CAt groups, there was an increase in the CML level by 14.5% in the well-controlled blood glucose group, while the CML level increased by 113% in the partially controlled blood glucose group. SOD activity increased by 55% in the well-controlled blood glucose group, while SOD increased by 115% in the partially controlled blood glucose group. However, SOD activity was found to decrease in the poorly controlled blood glucose group. From three subgroups, SOD activity seemed to be influenced by RBG more than HbA1c. In the CAo-treated group with RBG < 200 mg/dL, SOD activity was induced as a defence mechanism against oxidative stress.

From this subgroup analysis, we concluded that there was an increase in CML production in condition of HbA1c > 7%. IL-1*α* level was also found to be higher in the condition of HbA1c > 7%. In accordance with the previous findings, SOD activity was significantly higher in the partially controlled blood glucose subgroup of the CAo + CAt group compared to that of the other groups. The result also showed that SOD activity increased as an antioxidant response to pro-oxidant increment (CML and IL-1*α*) in the partially controlled blood glucose subgroup of the CAo + CAt group.

Subgroup analysis of SOD activity in well and partially controlled blood glucose subgroups in the CAo + CAt group showed that it was increased and statistically significant. However, in Plo + CAt, there were insignificant increases in SOD which implied the importance of CA in the oral regiment to maximize SOD activity enhancement. Two animal studies, specifically in the central nervous system of mice, found that the CA extract prevented the reduction in SOD in diabetes-induced mice. Both studies found that SOD activity was significantly higher in the CA-treated group [21, 22]. The animal study showed that CAo was effective in improving SOD activity in DM-induced mice where there was a reduction in SOD activity compared to the control. Correspondingly, our study found enhancement of SOD activity in T2DM patients treated with CAo + CAt. As the elevation of SOD activity in the partially controlled blood glucose subgroup was higher compared to the well-controlled blood glucose subgroup, we presumed that higher HbA1c promoted pro-oxidant activity that had to be countered by the antioxidant activity. In one correlation study comparing SOD activity in the HbA1c group (<7, 7–9, and >9), the SOD activity was higher in the DM patient compared to the normal. DM group with HbA1c < 7 had a higher significant SOD activity compared to the other groups [23]. Nonetheless, Doddigarla et al. [24] reported a contrary result where the patient with T2DM presented with low SOD activity and there was a correlation between decreased SOD activity and increased glycemic index.

In CAo + CAt treatment, CML in well-controlled blood glucose subgroup had the lowest increase and IL-1*α* increased less in well-controlled compared to the partially controlled and decreased in poor-controlled blood glucose. However, the analysis exhibited insignificant difference across group treatment. Previous studies showed CA lowered CML and IL-1*α*. In an in vivo study in the human explant, the CA extract inhibited formation of CML induced by methylglyoxal. The study also found significant IL-1*α* reduction in the CA extract treatment group after invoked by UV irradiation [25]. An in silico study of the CA compound showed that CA bound with IL-1*α* led to an inhibition [26]. A study of proinflammatory cytokines in human peripheral blood mononuclear cell (PBMCs) inhibition by the herbal methanol extract found that the CA extract inhibited IL-1*α* as much as 23.7 ± 4.57% in a 24-hour period. Compared to other herbal extracts in the study, inhibitory effect by CA still was less significant [27]. Decreased IL-1*α* in poorly controlled blood glucose in our study was similar to that of a study by Cantuaria et al. [17] where IL-1*α* decreased in hyperglycemia condition induced by *D-glucose* and lipopolysaccharide.

This study showed that SOD activity was responsive with controlled blood glucose both in HbA1c ≤ 7 and RBG < 200. The authors concluded that in controlled RBG < 200, antioxidant capacity in the group that received CAo was faster and more responsive to increase compared to groups who did not received CAo. Moreover, in this condition, antioxidant capacity will be expressed to cope with oxidative stress that is shown by the CML and IL-1*α* value. After 29 days of consuming CAo, antioxidant capacity has still not been able to reduce oxidative stress optimally; therefore, CML and IL-1*α* values were not lower compared to the placebo group. The author assumed that more than 29 days is necessary in resolving oxidative stress that is shown by the reduction in AGE formation seen in the decrease in the CML value and inflammation in IL-1*α*. More research studies are needed to explore this hypothesis.

From the result of this study, there was a strong correlation between CML with SOD activity and IL-1*α* with SOD activity in the CAo + CAt group well-controlled blood glucose subgroup. Therefore, we theorized that in the CAo + CAt group, SOD activity was more responsive in coping with oxidative stress represented by CML and IL-1*α*. In the CAo + CAt group, increased value of CML and IL-1*α* correlated with the increased SOD activity. However, this correlation did not happen in the other 2 groups. The intervention of CAo after 29 days in the CAo + CAt group had already induced the increase in SOD antioxidant capacity to be more responsive compared to the other 2 groups. Pieme et al. [28] found that uncomplicated DM and complicated DM patients have significantly more antioxidants compared to non-DM patients. This study also found that the pro-oxidants, which are MDA and nitrite oxide (NO), were significantly higher in DM patients compared to non-DM patients.

**Figure 9:**
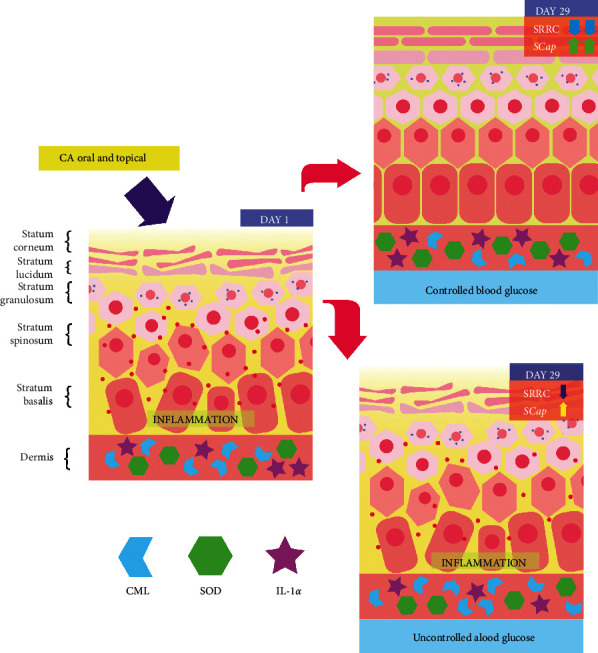
Comparison of the mechanism of CAo + CAt in improving dry skin in T2DM patients (controlled blood glucose and uncontrolled blood glucose patients).

We illustrate the mechanism of CAo + CAt treatment in improving dry skin in T2DM patients in Figure 9. In controlled blood glucose patients, the SOD activity will increase in response to the increase in CML and inflammation (IL-1*α*). In this condition, the capacity of the stratum corneum to hold water (SCap) will also ameliorate, leading to the improvement in clinical condition (SRRC score). Oral CA helps lowering blood glucose and providing glycation effects which can further lower CML production. In addition, CA also inhibits IL-1*α* activity. In conclusion, CAo + CAt is hypothesized to decrease inflammation response through the antiglycation effect by lowering CML production, antioxidant properties by increasing SOD activity, and inhibition of the IL-1*α* activity.

Oral and topical adverse events that occurred in the CAo + CAt group were not statistically significant compared to those in the Plo + CAt and Plo + Plt groups. A commonly complained adverse event was the gastrointestinal problem. The oral placebo contained amylum and magnesium stearate. Magnesium stearate functioned as a lubricant component and can break down into stearic acid, magnesium, and palmitate that are known to be safe for the human body. Magnesium had been known to cause gastrointestinal complaints and diarrhea [29]. This might explain the adverse events in the placebo group. The topical placebo used in this study was vaseline album.

## 5. Conclusions

In the well-controlled blood glucose subgroup, improvement of SSRC and SCap in the CAo + CAt group was better than that in the Plo + Plt group. In the partially controlled blood glucose subgroup, increment of SOD in the CAo + CAt group was higher than that in the Plo + Plt group. There was a moderate-to-strong correlation between CML or IL-1*α* and SOD activity in the well-controlled blood glucose subgroup. There were no significant adverse events in the CAo + CAt group compared to other two groups in the study. Therefore, the combination CAo and CAt on dry skin in T2DM patients is proven to significantly improve the outcome effectively and safely in controlled blood glucose HbA1c ≤ 7 and RBG < 200; thus, it can be recommended for the treatment of dry skin in T2DM patients.

## Figures and Tables

**Table 1:** Demographic, clinical, and laboratory characteristics based on the intervention group (*N* = 159).

Variable	CAo + CAt (*n* = 53)	Plo + CAt (*n* = 53)	Plo + Plt (*n* = 53)	*p* value^+^
Age (years)				0.04^*∗*^
Median (min–max)	52 (34–58)	54 (26–59)	53 (34–59)	
Gender, *n* (%)				0.97
Male	13 (24.5)	14 (26.4)	12 (22.6)	
Female	40 (75.5)	39 (73.6)	41 (77.4)	
Dry skin treatment history, *n* (%)	19 (35.8)	13 (24.5)	13 (24.5)	0.33
Pruritus VAS, *n* (%)				0.67^*∗*^
Not pruritus	39 (73.6)	43 (81.1)	39 (73.6)	
Low pruritus	14 (26.4)	9 (17)	14 (26.4)	
Moderate pruritus	0 (0)	1 (1.9)	0 (0)	
Dry skin duration, months				0.38^*∗*^
Median (min–max)	12 (0–360)	12 (0–420)	12 (0–480)	
BMI median (min–max) (kg/m^2^)	25.8 (18.8–38.2)	26.6 (19–39.8)	26.3 (20.4–35.3)	0.82^*∗*^
Hypertension, *n* (%)	30 (56.6)	33 (62.3)	33 (62.3)	0.79
Atopy history, *n* (%)	43 (81.1)	42 (79.2)	36 (67.9)	0.23
DM medication types, *n* (%)				
1 type OHA	23 (43.4)	25 (47.2)	23 (43.4)	0.90
2 types OHA	25 (47.2)	22 (41.5)	26 (49.1)	0.72
3 types OHA	3 (5.7)	3 (5.7)	3 (5.7)	1
Insulin	14 (26.4)	16 (30.2)	9 (17)	0.27
HbA1c (%)				0.33^*∗*^
Median (min–max)	7.7 (5.2–13.3)	8.95 (5.5–14.9)	7.4 (5–13.8)	
Triglyceride (mg/dL)				0.14^*∗*^
Median (min–max)	144 (62–616)	183 (64–1435)	173.5 (69–708)	
RBG (mg/dL)				
D 1				0.18^*∗*^
Median (min–max)	172 (69–444)	200 (66–473)	159 (77–524)	
SGOT (U/L)				
D 1				0.62^*∗*^
Median (min–max)	18 (9–48)	16 (8–48)	17 (10–68)	
SGPT (U/L)				
D 1				1.00^*∗*^
Median (min–max)	21 (7–70)	20 (6–57)	21 (6–88)	

Pearson chi-square. ^*∗*^Kruskal–Wallis test was performed to assess the difference. BMI = body mass index, OHA = oral antihyperglycemic agent, RBG = random blood glucose, SGOT = serum glutamic oxaloacetic transaminase, and SGPT = serum glutamic pyruvic transaminase.

**Table 2:** SRRC, SCap, CML, IL-1*α*, and SOD activity based on the intervention group (*N* = 159).

Variable	CAo + CAt (*n* = 53)	Plo + CAt (*n* = 53)	Plo + Plt (*n* = 53)	*p* value
SRRC				
D 1				0.07
Median (min–max)	4 (3–10)	4 (3–8)	5 (3–8)	
D 15				0.48
Median (min–max)	2 (0–6)	3 (0–7)	3 (0–7)	
D 29				0.71
Median (min–max)	2 (0–6)	2 (0–7)	2 (0–6)	
SCap (AU)				
D 1				0.34
Median (min–max)	27.7 (10.4–36.4)	25.4 (10.7–37.8)	26.2 (12.1–46)	
D 15				0.38
Median (min–max)	37.6 (14.5–61.6)	35.9 (12.5–61)	35 (16.1–59.9)	
D 29				0.39
Median (min–max)	43.5 (14.5–65.9)	44.6 (13.1–64)	38.5 (16.9–63.7)	
CML (pg/mg protein)				
D 1				0.79
Median (min–max)	87.2 (20.12–14559.42)	77.2 (4.1–385.7)	93.9 (14–407.8)	
D 29				0.41
Median (min–max)	119.8 (24.2–615.9)	119.4 (25.2–1731.4)	104.8 (22–748.1)	
IL-1*α* (pg/mg protein)				
D 1				0.60
Median (min–max)	16.5 (2.9–167.3)	16 (2.1–110.5)	17.6 (4.4–114.6)	
D 29				0.68
Median (min–max)	19.7 (3.2–167.3)	18.2 (4.9–69.6)	17.6 (4.9–114.5)	
SOD (U/mg protein)				
D 1				0.31
Median (min–max)	4.6 (0.3–59.4)	3.4 (0.3–41.5)	3.9 (0.2–35)	
D 29				0.07
Median (min–max)	5.9 (1–59.4)	4.3 (0.2–18.7)	4.9 (0.1–23.6)	

Kruskal–Wallis test was performed to assess the difference.

**Table 3:** Comparison of SRRC, percentage of SRRC decrement, and SCap subgroup analysis in well-controlled blood glucose subjects.

Parameter	CAo + CAt	Plo + CAt	Plo + Plt	*p* value
SRRC				
D 1				0.14
*n*	18	16	21	
Median (min–max)	4 (3–10)	5 (3–7)	5 (3–8)	
D 15				0.77
*n*	20	18	18	
Median (min–max)	2 (0–6)	2.5 (0–4)	2 (0–7)	
D 29				0.12
*n*	20	17	18	
Median (min–max)	1 (0–5)	2 (0–5)	2 (0–6)	
Percentage of SRRC decrement				
D 15				0.55
*n*	20	18	18	
Median (min–max)	50 (0–100)	50 (0–100)	33.3 (0–100)	
D 29				0.11
*n*	20	17	18	
Median (min–max)	**75** (16.7–100)^a^	60 (0–100)	**50** (14.3–100)	
SCap (AU)				
D 1				0.30
*n*	18	16	21	
Median (min–max)	27.9 (15.6–36.4)	24.3 (15–32.7)	25.9 (13.8–46)	
D 15				0.36
*n*	20	18	18	
Median (min–max)	40.5 (21.1–51.6)	38.1 (21.8–58.5)	36.1 (21.5–59.9)	
D 29				**0.03**
*n*	20	17	18	
Median (min–max)	**47** (30.7–61.3)^a^	**45.6** (20.1–15.5)	**38.5** (19.7–53.4)	

Kruskal–Wallis test amongst three groups, followed by the Mann–Whitney test, to assess the difference between each group. ^a^Compared to Plo + Plt with *p* < 0.05.

**Table 4:** Comparisons of CML, IL-1*α*, and SOD analysis in the partially controlled blood glucose subgroup.

Parameter	CAo + CAt	Plo + CAt	Plo + Plt	*p* value
D 1				
*n*	13	9	14	
CML (pg/mg protein)				0.55
Median (min–max)	73.9 (26.7–219.9)	153 (35.5–179.6)	70.9 (27.5–279.4)	
IL-1*α* (pg/mg protein)				0.67
Median (min–max)	17.3 (7–32)	17.9 (4.4–96.2)	16.7 (6.8–47.1)	
SOD (U/mg protein)				0.28
Median (min–max)	3.9 (0.3–11.2)	6 (0.4–25.3)	2.7 (0.2–16.4)	
D 29				
*n*	13	7	14	
CML (pg/mg protein)				0.42
Median (min–max)	158 (26–524.6)	82.7 (44.3–270.1)	91.8 (22–422.1)	
IL-1*α* (pg/mg protein)				0.51
Median (min–max)	21.6 (7.9–65.9)	17 (6.2–32.2)	14.5 (4.9–80)	
SOD (U/mg protein)				**0.03**
Median (min–max)	8.4 (1.3–25)^**a**^	2.4 (1.3–10.5)	3.5 (0.2–8.1)	

Kruskal–Wallis test amongst the three groups, followed by the Mann–Whitney test, to assess the difference between each group. ^a^Compared to Plo + Plt with *p* < 0.05.

## Data Availability

The data used to support the findings of this study are available from the corresponding author upon request.
